# Factors influencing adherence to regular exercise in middle-aged women: a qualitative study to inform clinical practice

**DOI:** 10.1186/1472-6874-14-49

**Published:** 2014-03-26

**Authors:** Deanne McArthur, Alex Dumas, Kirsten Woodend, Sarah Beach, Dawn Stacey

**Affiliations:** 1 School of Nursing, Faculty of Health Sciences, University of Ottawa, 451 Smyth Road, Room RGN 1118, Ottawa, Ontario K1H 8M5, Canada; 2 School of Human Kinetics, Faculty of Health Sciences, University of Ottawa, 125 University Private, MNT 366, Ottawa, Ontario K1N 6 N5, Canada; 3 Trent/Fleming School of Nursing, Trent University, 1600 W Bank Dr, Peterborough, ON K9J 7B8, Canada; 4 Centre for Practice-Changing Research, Clinical Epidemiology Program, Ottawa Hospital Research Institute, 501 Smyth Road, Box 201B, Ottawa, Ontario K1H 8 L6, Canada

**Keywords:** Women, Middle-life, Physical activity, Barriers, Qualitative study, Enablers

## Abstract

**Background:**

About half of women decrease their regular exercise during middle age. Concurrently, they experience a reduction in basal metabolic rate and loss of lean muscle as they transition to menopause. The combined effects place these women at increased risk for body weight gain and associated co-morbidities. Further research is required to better assess their barriers to regular exercise and to develop more applied knowledge aimed to improve the applicability of clinical interventions aimed at this population. The main aim of this study was to identify enablers and barriers influencing adherence to regular exercise in middle-aged women who exercise.

**Methods:**

An interpretive description qualitative study was conducted using individual interviews. The two key questions were focused on planning to engage in physical activity and succeeding or planning to engage in physical activity and not succeeding. Inductive content analysis was used.

**Results:**

Fifty-three women interviewed were aged 40–62 years and experiencing mild to moderate menopausal symptoms. Six broad themes influencing adhering to regular exercise were: routine, intrinsic motivation, biophysical issues, psychosocial commitments, environmental factors, and resources. Common sub-themes were identified as enabling factors: daily structure that incorporated physical activity (broad theme routine), anticipated positive feelings associated with physical activity (intrinsic), and accountability to others (psychosocial). Other common sub-themes identified as barriers were disruptions in daily structure (routine), competing demands (routine) and self-sacrifice (psychosocial).

**Conclusions:**

The most common barrier middle-aged women describe as interfering with adhering to regular exercise was attributable to the demands of this life stage at home and with others. Lack of time and menopausal symptoms were not identified as the common barriers. To support women to adhere to regular exercise, healthcare professionals should consider a narrative approach to assessing barriers and focus on enablers to overcoming identified barriers.

## Background

Women significantly reduce regular exercise during middle-age by up to 40% [1-3]. Concurrently, women experience a reduction in basal metabolic rate and loss of lean muscle as they transition to menopause [4]; thereby increasing their risk for body weight gain and obesity. Obesity is associated with co-morbidities including diabetes, hypertension, cardiovascular disease, and some cancers [5].

Interventions to prevent or reduce weight gain include regular exercise combined with healthy eating [6,7]. Clinical practice guidelines for adults recommend an energy reduced diet, regular exercise (with increased exercise for weight loss), education and support in behaviour modification, cognitive behavioural therapy, and dietary counselling [8]. One large randomized trial showed that expending 1000–1500 kcal/week (equivalent of brisk walking 10–15 miles/week) and consuming a healthy diet of 1300 kcal/day was an effective intervention for middle-aged women as they transitioned through to menopause [7]. However, factors interfere with engaging in exercise.

Common barriers for middle-aged women considering initiation of exercise are lack of time, safety concerns about exercising outdoors, weather, and not having a family member or friend to exercise with [9]. Lack of time is further attributed to multiple responsibilities and roles of women within their households and at work [10]. Although these barriers are known to discourage the decision to engage in regular exercise, little is known about what factors interfere with middle-aged women adhering to a regular exercise program once they have decided to do so. Adherence is defined as an individual’s trajectory from making a positive lifestyle choice, in this case, to exercise through to implementing the necessary steps to carry out the plan of action [11]. Although there are systematic reviews of adherence to dietary interventions for individuals with chronic illnesses, adherence to medications, and adherence to group exercise to prevent falls in older adults [12-14], there is no known review of adherence to regular exercise among middle aged women.

Encouraging uptake of regular exercise is preventative healthcare and common practice in primary care. In a survey of 398 nurse practitioners, half indicated that they had counselled about half of their patients within the previous week to exercise and most agreed that exercise counseling was as important as prescribing medications [15]. However, most research is focused on exercise counseling to increase adherence in older adults, individuals with chronic conditions, or children [16-19] and not healthy middle-aged women [13,15].

The purpose of this study was to identify enablers and barriers influencing adherence to regular exercise in middle-aged women who exercise. The study took place within a team grant “Sherbrooke-Ottawa-Montréal Emerging Team (SOMET) on Critical Periods of Body Weight Regulation: A Women’s Health Perspective” within a larger mixed methods descriptive study exploring individual and environmental factors influencing 60 middle-aged women implementing decisions for achieving and maintaining healthy body weight. This study is an analysis of qualitative data which focused on these women’s experiences with adhering to regular exercise.

## Methods

### Study design

An Interpretive Description qualitative approach was used. Interpretive description design was developed to gain an understanding of a phenomenon that would facilitate knowledge development to inform clinical practice [20,21]. This qualitative approach was chosen to guide the present study due to its alignment toward the generation of clinically-relevant knowledge [20]. Study approval was received from the University of Ottawa Research Ethics Board (H03-10-07).

### Setting and participants

Eligible participants were middle-aged women, 40 to 65 years old and living in an urban Canadian city with a population of about 800,000.Women were recruited from the community through posted materials in local neighbourhoods and through free online classified advertisements. For this study, a sub-set of women from the larger study about body weight decisions were included if they indicated that they participated in any regular exercise in an average week. Examples of regular exercise included walking, organized sports, and/or independently structured exercise. Women from the larger study were ineligible if they indicated that they did not engage in any regular physical activity during an average week or the audio-files of their interview were inadequate for analysis.

### Data collection

Women were interviewed by a research assistant (SB, AmD) after signing a consent form. Interviews took place at a convenient location and time using a structured interview guide. For this sub-analysis, women who indicated that they participated in regular physical activity during an average week were asked to describe the type of physical activity. Then, participants were asked two key questions:

● Think about a time when you incorporated physical activity into your day. Tell me about your initial thoughts of exercising on that particular day all the way through to the actual execution of doing that exercise.

● Now think of a time you planned to incorporate physical activity into your day, but for some reason or another you were unable to. Tell me about your initial thoughts of exercising on that particular day all the way through to the actual point when you were unable to exercise.

The study questions were pilot tested with five participants within the larger study. Participants had no difficulty with the questions asked and given that no changes were made their interviews were retained for analysis. All interviews were audio-recorded.

Demographic information included age and education level. Women’s reported body weight and height was used to calculate their body mass index and women reported whether or not they felt safe walking in their neighbourhood in the day and at night. Severity of menopausal symptoms was also measured using the validated Menopausal Rating Scale [22]. This scale asks women to rate their psychological, somatic, and urogenital menopause-related symptoms on a scale from 0 (no symptoms) to 4 (very severe symptoms).

#### Data analysis

Digital recordings of the interviews were transcribed verbatim. Following the principles of Interpretive Description [20], analysis was conducted independently by two team members (DM, SB). First, the written transcriptions were reviewed while listening to the audio recordings to ensure accuracy of the transcripts and determine themes that emerge inductively from an ‘aerial view’ of the data. Transcripts were analyzed line by line for themes reflecting factors affecting middle-aged women’s adherence to regular exercise. Comparative analysis was conducted with subsequent transcripts to build findings upon themes that had previously emerged. Consistent with the Interpretive Description approach [20], themes were first subject to broad inclusion so as not to restrict the validity of the data due to premature categorization. As further interviews were analyzed, responses were grouped first into sub-themes, with those eventually being clustered under applicable broad themes. Demographic data was entered into an Excel database and analyzed descriptively.

## Results

### Characteristics of participants

Of 60 women interviewed between June 2010 and December 2011 in the larger study, 53 indicated that they participated in regular physical activity on an average week, 5 do not participate in regular physical activity, and 2 were excluded due to inadequate interview recordings. For the 53 women, physical activity was described as inconsistent (n = 3), walking only (n = 19), or involving multiple activities (e.g., walk, run, cycle, swim, weight training, dance, yoga, ski) (n = 31) (see Table 1).

**Table 1:** Characteristics of the participants

**Characteristics**	**Participants (N = 53)**
**Age(years)**	
40-45	12
46-50	13
51-55	17
56-60	9
61-65	2
**Education**	
Secondary school	2
Some college	5
College	12
Some university	7
University	27
**Types of physical activity**	
Multiple activities (e.g., walk, run, cycle, swim, weights, dance, yoga, ski)	31
Walking only	19
Inconsistent	3
**Menopause severity score**	
0 none	1
1-10	18
11-20	20
21-30	13
31-40	1
40-44 very severe	0
**Menstrual periods**	
None for 1 year or longer	25
Yes within last year	28
**Body mass index**	
18.4 or lower	3
18.5 to 24.9	19
25.0 to 29.9	15
30.0 to 39.9	13
40.0 or higher	3
**Feel safe to walk in the neighbourhood**	
In the day	51
After night	31

N/R = not reported.

Women’s body mass index ranged from being lower than 18.5 to higher than 40 (mean 28). Most women felt safe walking in their neighbourhood during the day and more than half at night. Women were aged 40 to 62 years (mean = 50) and their level of education ranged from secondary school to university. Half of the women menstruated within the last year (n = 28) and the other half did not (n = 25). Fifty-two experienced menopausal symptoms that ranged from mild to moderate and one woman experienced no menopausal symptoms.

### Factors influencing adherence to regular exercise

**Figure 1:**
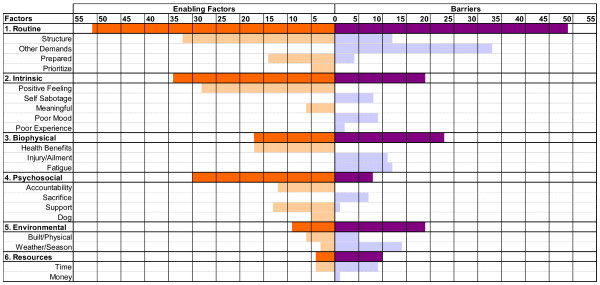
Themes and sub-themes of enablers and barriers influencing middle aged women adhering to their regular exercise.

Six broad themes on enabling factors or barriers for adhering to regular exercise were identified: routine, intrinsic motivation, biophysical issues, psychosocial, environmental factors, and resources. The themes are discussed from most common to least common (see Figure 1). Additionally, each broad theme was divided into sub-themes for a total of 20 sub-themes.

### Routine

The extent to which women integrated exercise into their routine was characterized by four sub-themes: exercise incorporated into daily structure, other demands, prepared, and prioritized (see Figure 1). ‘Exercise incorporated into daily structure’ reflected women’s ability and inclination to organize their daily lives to include exercise. For example, *“… it was Monday, Wednesday, Saturday … those were the days that we already planned and we would go…*”. Conversely, the interruption of this structure was viewed as a barrier: *“… I didn't do it [exercise] yesterday because I had an appointment early*”. Other demands’ was the most significant barrier reported by the women in their attempts to uphold their intentions to be physically active: *“… the bike ride got pushed over because I had to madly clean the house ..*.”. ‘Being prepared’ emerged as the third sub-theme and refers to having the necessary equipment (e.g. gym clothes) and arrangements in place ahead of time. Being prepared was viewed as an important enabling factor, while being unprepared was viewed as an important barrier: *“… I made sure I had all my gym stuff with me when I went to work, and that’s something where [if] I’ve forgotten it, I say: well, I can’t go anyways*”. ‘Prioritized’ depended on the emphasis women placed on exercise relative to other aspects of their daily lives: *“… work gets in the way … but I know, because I'm committed, that I'll go the following day*”.

### Intrinsic motivation

‘Intrinsic motivation’ refers to the inherent satisfactions women experience from physical activity including feelings of enjoyment and accomplishment [23]. These psychological factors influence adherence to regular exercise. There were five sub-themes. ‘Anticipated positive feelings’ included physiological sensations (e.g. a “rush” during and/or following exercise), or emotional (e.g. a feeling of accomplishment): *“… I know the end result is always a good feeling*”. ‘Self sabotage’ was evident when women described putting obstacles in the way to avoid exercise, even though they set out to be active. For example,

“I’ll go do groceries, and I’ll stay in my outfit. The grocery store has the gym in front of it and I’ll say: I’ll do the exercise first. But as I’m walking toward the grocery store … I’ll say: no, I’ll go after I do the groceries, cause I’m worried about the specials – that I won’t get them … and then I look at all these bags that I’ve paid for, and I say: if I bring them out to the car now … I’ll go to the gym after – I’ll just drop these in the car … Then I’m saying: no, I have frozen products and I can’t go to the gym – so what I’ll do is I’ll bring the stuff home. So, this goes on all day, and then I’ll get home, I’ll put all the stuff in the freezer…and then I’ll say to myself: oh, this outfit – I’m not comfortable – let me get out of it … And then I’m screwed, right? I’m out of my outfit. … And then I’ll eat. Then I’ll be like: I’m too full – I’ll go in my sweat pants. And the more – the lies to myself – and then, then I’m in my sweatpants, and I’ll say: well I can have a chocolate bar now, you know, and then I’ll go. Right? … And then it just gets worse and worse, cause then I’ll say: well, you know, I’m overweight – I shouldn’t have eaten that chocolate bar”… it’s insane”.

‘Meaningful Exercise’, refers to the preference for seeking novel or purposeful opportunities that deviated from the regimented routines of gym workouts: “… *when I have a reason and an activity I enjoy, it's much easier to be physically active*”. ‘Negative/ambivalent mood’ captures a range of emotional responses to exercise, from ambivalence to more profound feelings such as frustration or even depression. While some participants noted the mitigating effect of exercise on these feelings, responses were attributed to this category when women stated that feelings were a barrier to participation: “*Well, if I wake up in the morning and I'm in a bad mood, and … I say to hell about the gym*”. ‘Poor experience’ captures the detrimental effects of prior negative experiences either in terms of their physical performance (e.g. frustration with perceived lack of ability) or emotional upset (e.g. a gym employee labelling a respondent “obese”).

### Biophysical considerations

Women identified perceived health benefits as an enabling factor with injury and fatigue as barriers within the broad theme of ‘biophysical considerations’ or physical wellbeing. ‘Perceived health benefits’, pertains to the motivation attached to exercise as a contributing factor to overall health. For example, one woman said: “*I find it [exercise] really relieves stress…*”. ‘Injury/ailment’ captures the impact of either acute or ongoing physical constraints on their exercise: *“… my physical activity has been compromised because I have a back and ankle situation*”. ‘Fatigue’ captured responses wherein women articulated simply being “too tired” to be active: “… *when I get home, I don’t feel like it anymore, cause I'm too tired*”.

### Psychosocial

There were four sub-themes identified as enablers and/or barriers within the broad theme of ‘Psychosocial’. This theme refers to interactions between the participants and others that either encouraged or discouraged exercise. ‘Accountability to others’ referred to the degree to which the women felt beholden to others (either through mutual participation or awareness of intent) to keep to their exercise commitments. For example, one woman said *“… now I really can't bail cause everybody knows I'm going*”. ‘Self-sacrifice’ was a barrier in which women forewent their exercise in order to attend to the needs or wants of others. As one woman described *“… planning a time [for exercise], but then something happens … somebody needs something … and I just can't fit it in*”. ‘Support’, refers to the way in which others, through verbal encouragement, organizational help (e.g. childcare), or social interaction enable the women to be active. An example of support as an enabling factor was *“… it's the support of my husband … he knows how important it [exercise] is to me … he's super, super supportive … and that means a lot to me*”. ‘Feeling responsibility to a dog’ was an enabling factor. One woman described buying a dog so that her choice to exercise would be mandated: “*… I think: I have to walk the dogs … I have to because I don't have a choice, which is a good thing*”.

### Environmental

The broad theme ‘environmental’ refers to women’s physical surroundings for which two sub-themes emerged. ‘Built environment’ was described in terms of how the infrastructure surrounding the women’s work/home allowed for safe integration of exercise into transportation or leisure activity: *“… my transportation then was bicycle. I was in Montreal and that worked well … it’s not as bike friendly in Ottawa as it is in Montreal…”*. The bi-directionality of the ‘weather/climate’ sub-theme was captured by the following quote: *“… it was a beautiful day out … and I just said: ok-get up and go … I'm not sure what we'll do in the winter, like the icy days*”.

### Resources

Within the sub-theme ‘resources’, a few women identified time or money as factors influencing adherence to regular exercise. Some women described middle-age as a point in their lives where they had more time to devote to exercise, while others reported the opposite: “*That's pretty much all the time I have to exercise … because I'm a full time employee and I was also a full time student at the same time the past year*”. Another woman discussed ‘lack of money’ as a barrier to being physically active: *“… if you don't have money you can't join a gym or body building place …*”.

## Discussion

Exercise patterns of middle-aged women represent a complex intersection of internal and external factors. The broad themes identified as influencing women’s adherence to regular exercise were routine, intrinsic motivation, biophysical, psychosocial, environmental, and resources. Within each of these broad themes, the sub-themes were identified by the women as either enablers, barriers, or both. Interestingly, although most women were experiencing menopausal symptoms, they were not identified as a factor affecting women’s adherence to regular exercise. Rather, the majority of factors were related to psychosocial aspects of this mid-life stage. Unlike findings of other studies, time and environmental issues were not identified as common barriers in our study [24,25]. This could be attributed to the way in which we specifically asked women to tell us a story of a positive and negative experience about adhering to exercise rather than just identify their perceived barriers or enablers. The fact that climate was not of concern was surprising given we conducted the research in the variable and often extreme Canadian climate. Consistent with barriers to exercise within biophysical factors, other studies have shown that illnesses can trigger concern for health and the adoption of an active lifestyle [26].

### Factors enabling women’s participation in regular exercise

The most cited factors enabling adherence to their regular exercise were: an established daily structure that incorporated exercise (broad theme routine), anticipated positive feelings associated with exercise (intrinsic motivation), and accountability to others (psychosocial). The greater the degree to which the women were able to achieve automaticity within their daily structure, the less susceptible exercise was to being replaced by other priorities. As consistency with maintaining participation in exercise is strengthened, the activity is more likely to become a “habit” and become an intrinsic part of a woman’s daily routine [27,28]. The theme of anticipated positive feelings and women’s desire to replicate those feelings was often described as sufficient motivation for them to schedule exercise and/or overcome incidental barriers.

The feeling of achievement the women derive from not only having adhered to their “good” intentions to exercise, but also from continued, improved exercise competence, prompted their continued participation. The emergence of this relevant sub-theme coincides with Jeng’s description of “perceived continuous power”; overcoming psychological and physical discomfort of initiating exercise to experiencing benefits and making new friends who exercise [29]. Another important enabler was accountability to others which could be capitalized upon to facilitate conditions of enhanced social support [30].

### Barriers to women’s participation in regular exercise

The three most prevalent barriers were disruptions in daily structure (routine), competing demands (routine) and self-sacrifice (psychosocial).Women discussed the challenges they face in their efforts to preserve their commitment to exercise in light of the other, often competing demands placed upon their time, energy and resources. While ‘competing demands’ is described separately from disrupted daily structure, the two overlap to produce a situation wherein the activities women had planned for their day could become vulnerable to competing interests both internal and external to themselves. Typical conflicts within this sub-theme existed between obligations the women had to themselves (e.g. medical appointments) or those they perceived in response to others (e.g. cleaning the house in preparation for guests). This theme aligns well with earlier studies that showed women’s participation in exercise is affected by the multiple responsibilities they carry at home and at work [31]. Two barrier sub-themes of ‘intrinsic motivation’ (i.e. ‘negative/ambivalent mood’ and ‘self-sabotage’) captured the confluence of cognitive and emotional elements underlying the women’s decision to exercise. Their actions seemed to demonstrate a response to their emotional state (“I don’t feel like going to the gym”) as it conflicts with their cognitive appraisal (“I should exercise”). The resulting self-sabotage is an attempt to justify their ambivalence overtaking their intention. Healthcare professionals can facilitate recognition and exploration of this emotional influence.

### Implications for clinical practice

The primary value of this qualitative study was that it allowed women to provide narratives, in their own words, of factors that hinder or enable their adherence to regular exercise regardless of their intent. Armed with this knowledge, healthcare practitioners are better poised to move therapeutic conversations beyond knowledge delivery (e.g., you need to exercise more) to those factors that are likely to be relevant to adhering to regular exercise. Given that these enablers and barriers are multi-factorial, clinical interventions should be framed within a more holist approach (see Table 2). First, healthcare professionals should go beyond assessing barriers to exercise and focus middle-aged women on ways to overcoming barriers. Addressing barriers and motivators are key elements in motivational interviewing to help women achieve their established goal (e.g. to engage in regular exercise) [32]. Two enablers motivating women in our study were positive feelings and perceived benefits to health. Participation in exercise has been a strong predictor of the health for women during and following midlife [33]. However, there is the relationship between factors that ultimately result in an increase, maintenance, or decline in levels of participation. These factors go beyond inadequate knowledge to include social, emotional, and other psychological influences [33]. Particular attention should aim to identify the range of factors that can influence middle-aged women and help them find ways to overcome their own barriers to regular exercise adherence [9,10,15,34].

**Table 2:** Actions for promoting adherence to regular exercise in middle-aged women

1.	Assess psychosocial context of women’s lives.
2.	Go beyond encouraging regular exercise to explore factors influencing women’s adherence to exercise (e.g. using narratives).
3.	Encourage women to plan regular exercise within their weekly schedule.
4.	Suggest women identify others to exercise with.
5.	Collaborate with other healthcare professionals, as necessary, to ensure a comprehensive approach.

An important goal among healthcare professionals is to encourage clients to make healthy lifestyle choices, such as engaging in exercise. Priority appeared to be a buffer to would-be barriers that women described in our study. For example, one woman spoke of her ability to reschedule her exercise when other obligations took place during her allotted time. The manifestation of prioritization to ensure adherence to exercise seems to represent the ultimate balance against barriers. While knowledge of exercise as an important aspect of health may inform prioritization, the findings of the present study suggest that the process is more complex, and that healthcare interventions limited to the knowledge aspect of prioritization are incomplete.^9^ Women already perceive that they “should” be more active [35]. An intervention for healthcare professionals, then, is to assist women in translating that conviction into a positive motivating factor as opposed to a source of frustration. For exercise to evolve to an experience that achieves anticipated positive feelings, women must engage in self-reflection in order to determine what sort of activities are likely to produce such an experience for them [36].

### Strengths and limitations

To enhance the credibility of findings, the interviews took place in natural settings, the time and place of which were determined by the participant. Interview transcriptions were quality-checked during the analysis. Credibility of the present findings was strengthened through data analysis conducted by two team members independently and reaching agreement by consensus [37]. Dependability was ensured during data collection by adherence to the interview script and by maintaining a detailed audit trail [37]. By conducting this qualitative study within the broader context of the SOMET grant was the ability to test the assumptions of saturation [38]. We were able to provide evidence that saturation reached by 10 interviews in the first set of 13 transcripts was stable with no further themes emerging with the subsequent set of 40 interviews. However, a limitation is the minimal detail on the frequency and intensity of physical activity of the women who participated. Despite efforts to recruit women with various personal characteristics, few participants had more severe menopausal symptoms. This may have influenced the finding that menopausal symptoms did not appear to influence engagement in exercise. However, the severity of menopausal symptoms experienced by women in our study were similar to other studies of middle-aged women [39,40].

## Conclusions

Middle-aged women’s adherence to regular exercise is the result of a complex interplay of social, emotional, environmental, and psychological factors. Neither menopausal symptoms nor knowledge deficit appeared to play a significant role in women’s adherence to regular exercise. The most common enabling factors influencing a woman’s adherence to regular exercise were having daily structures that incorporated exercise, anticipated positive feelings associated with exercise, and accountability to others. The most significant barriers were other demands, disrupted daily structure, and self-sacrifice. Further research should explore the factors influencing adherence to exercise in women of lower socio-economic status, evaluate social media interventions to enhance participation, and examine psychosocial influence on adherence. To support women to continue regular exercise, healthcare professionals should assess barriers and enablers that may be unique to these women. Using a narrative approach to assessing factors influencing their adherence to regular exercise and focusing on enablers may overcome known barriers within control of these women.

## Competing interests

The authors declare that they have no competing interests.

## Authors’ contributions

DM conceived this sub-analysis and led the research study including data analysis and writing the manuscript. DS participated in the conception of the study, data analysis, and writing of the manuscript. AD and KW participated in the conception of the study and writing of the manuscript. SB participated in the analysis of the data, and writing of the manuscript. All authors read and approved the final manuscript.

## Pre-publication history

The pre-publication history for this paper can be accessed here:

http://www.biomedcentral.com/1472-6874/14/49/prepub
